# Sex-Specific Relationship of Childhood Adversity With Gray Matter Volume and Temperament

**DOI:** 10.3389/fnbeh.2019.00071

**Published:** 2019-04-12

**Authors:** Wojciech Łukasz Dragan, Katarzyna Jednoróg, Artur Marchewka

**Affiliations:** ^1^Interdisciplinary Centre for Behavior Genetic Research, Faculty of Psychology, University of Warsaw, Warsaw, Poland; ^2^Laboratory of Language Neurobiology, Nencki Institute of Experimental Biology, Polish Academy of Sciences, Warsaw, Poland; ^3^Laboratory of Brain Imaging, Neurobiology Center, Nencki Institute of Experimental Biology, Polish Academy of Sciences, Warsaw, Poland

**Keywords:** childhood adversity, temperament, BIS, BAS, GMV

## Abstract

**Background**: To date, many studies have attempted to show a relationship between potentially harmful experiences in childhood and gray matter volume (GMV) in specific brain areas. These studies managed to identify several affected regions, yet most of them neglected the influence of sex or the occurrence of mental health problems. Furthermore, little is known about mechanisms linking childhood adversity (CA) and temperamental traits as plausible endophenotypes of psychopathology.

**Objective**: The present study addresses these two issues by trying to identify sex-specific relationships between CA and brain volumes as well as to show the role of the latter in predicting temperament scores.

**Method**: Forty-eight people (23 women) without anxiety or affective disorders participated in this study. CA was measured using the Childhood Questionnaire (CQ) and temperament was measured with the use of the behavioral inhibition system-behavioral activation system (BIS-BAS) Scales. Whole-brain MR imaging was performed to identify GMV differences.

**Results**: In women, we identified negative relationships between CA and GMV in the left inferior parietal lobule (IPL), right cerebellum, and right precentral gyrus. In men, we found a negative correlation between CA and GMV in the right fusiform gyrus. We also identified sex-specific relationships between CA and temperament traits.

**Conclusions**: The results of our study suggest a sex-specific pattern in the relationship between early adverse experiences and brain structure. The results can also help explain the role that temperament plays in the relationship between CA and the risk of psychopathology.

## Introduction

The concept of childhood adversity (CA) refers to a group of potentially harmful experiences such as emotional, sexual and physical abuse, neglect, parental loss, or illness which occur during childhood. Different childhood negative experiences have long been examined as risk factors for various psychiatric disorders (e.g., Catani and Sossalla, 2015). A growing body of evidence shows relationships between CA and gray matter volume (GMV) in specific brain areas. A recent whole-brain meta-analysis (Paquola et al., 2016) revealed reduced gray matter in the right dorsolateral prefrontal cortex (dlPFC), right hippocampus, and right postcentral gyrus among adults with a history of CA. These brain areas were also identified in some empirical articles: the right dorsolateral PFC by Carballedo et al. (2012) and Duarte et al. (2016), right hippocampus by Frodl et al. (2010) and right postcentral gyrus by Fonzo et al. (2013). Smaller volumes among CA survivors were also found in other brain regions such as the left inferior parietal lobule (IPL; Cheng et al., 2015), right and left cerebellum (De Bellis and Kuchibhatla, 2006), right precentral gyrus (Brooks et al., 2014), and visual cortex including right and left fusiform gyrus (Tomoda et al., 2009). Hart and Rubia (2012) indicate some limitations of research conducted in this area. First of all, they point to a lack of studies on sex differences. They suggest that it is possible that CA manifests differently in women and men due to different sex-specific trajectories of brain development. A growing body of data indicates sex differences in how early life stressors affect brain development. For example, Markham et al. (2013) used a rat model to show the relationship between prenatal stress and a sex-specific pattern of dendrite structure which was present in adolescent males, but not females. A recent study by Everaerd et al. (2016) showed an interaction between type of CA and sex—women who experienced either deprivation or abuse had less GMV in the visual posterior precuneus than women without any negative experiences, while men who experienced childhood deprivation had less GMV within the postcentral gyrus than those who had experienced abuse. Moreover, the meta-analysis of Paquola et al. (2016) revealed that both the right dlPFC and right postcentral gyrus showed significantly more reduction in cohorts with more females. These findings suggest that negative environmental conditions such as CA may have different sex-related neuronal consequences. This may be due to the impact of gonadal hormones or different “sensitive” time windows during neural development in both sexes (Crozier et al., 2014).

The main purpose of this study was to examine the potential association between CA and GMV in both sexes. Based on the results of the meta-analysis of Paquola et al. (2016), we particularly expected an association between CA and the GMV of the right dlPFC and right postcentral gyrus among women.

Hart and Rubia (2012) also indicated that a large number of studies on neuronal correlates of CA were carried out in abused individuals who also had associated psychiatric conditions. For example, Paquola et al. (2016) found differences in the GMV of the right dlPFC and right postcentral gyrus between healthy cohorts and groups with existing psychiatric conditions. Moreover, as a result of existing psychiatric problems, many participants in the aforementioned studies were treated with psychotropic medication. It is known that antidepressants and psychostimulants may affect brain structure and function (Kong et al., 2014; Rubia et al., 2014). For these reasons, it is hard to separate which structural differences are the result of CA and which are linked to current mental health. It has to be mentioned that some studies showed the relationship between differences in the GMV of brain areas and CA among subjects without psychiatric conditions. For example, Carballedo et al. (2012) revealed a reduction of dorsolateral prefrontal cortices, medial prefrontal cortices and anterior cortex cingulate in a group of healthy subjects with the history of emotional abuse. One must keep in mind however that the studies on the CA-GMV relationship using samples without a mental health diagnosis are scarce. As mentioned previously, CA is related to mental health problems in adulthood. It seems plausible to treat the neuronal correlates of childhood negative experiences as potential signs of risk of mental health problems. Thus, we believe that using groups without psychiatric conditions may help to unveil a clear pattern of neuronal correlates of CA.

Therefore, following the recommendations of Hart and Rubia (2012), our research objectives are implemented based on a group of subjects without current or lifetime diagnoses of mental health problems.

Another question raised in the current study is related to the psychological mechanisms mediating the relationship between CA and the risk of mental health problems. According to Strelau (2008), temperamental traits may serve as potential moderators of the stress response, and therefore this concept may be useful in explaining the relationship between CA and adult psychopathology. Few studies have shown a link between CA and temperamental or personality traits. Allen and Lauterbach (2007) revealed that individuals who were traumatized as children scored higher than controls in neuroticism and openness to experience (traits which are distinguished by the five-factor model of personality). Li X. et al. (2014) found that subjects with a high level of CA scored higher in neuroticism and psychoticism than those without such experiences. Interestingly, Sudbrack et al. (2015) found a sex-specific pattern of association between early experiences and temperament traits with men showing correlation with anger and women showing correlation with anxiety. This finding is in line with the results of some studies showing sex-specific patterns of relationships between CA and psychopathology. For example, Oldehinkel et al. (2008) found that parental divorce was related to depressive symptoms in girls, but not in boys, while Schilling et al. (2007) showed that men who experienced CA are more likely to engage in antisocial behaviors than women. Some studies, however, did not find any sex differences in CA and psychopathology associations (Oldehinkel and Ormel, 2014).

The first version of Gray’s (1982) model of temperament considered the existence of two specific neurophysiological mechanisms which can be measured at the behavioral level—the behavioral inhibition system (BIS) and the behavioral activation system (BAS). BIS is related to the trait-anxiety dimension and BAS is related to impulsivity and extraversion (Smillie et al., 2006). Among women, BIS activity is higher, as is the prevalence of anxiety and affective disorders, whereas BAS activity is higher among men, as is the prevalence of antisocial behavior (e.g., aggression) and impulsive behavior (e.g., ADHD; Boyd et al., 2015). Carver and White’s (1994) conceptualization of BIS and BAS includes three components of the latter: reward Responsiveness, which reflects positive responses to anticipation and reward; Drive, which is related to the tendency to persistently pursue desired goals; and Fun Seeking, which is the desire for novel rewards and willingness to approach potentially rewarding situations. Interestingly, neuronal correlates of differences in BIS and BAS activity have been identified. In particular, Li Y. et al. (2014) found sex differences in correlations between BIS and BAS scores and regional GMV. Taking into account all the findings mentioned above, an additional aim of this study was to identify sex-specific correlations of CA and BIS/BAS activity. Specifically, we hypothesize that CA will be related to BIS activity scores among women and to BAS activity scores among men.

## Materials and Methods

### Participants

A total of 48 people (23 women) aged 21–47 participated in the study (*M* = 25.1, SD = 5.36). The research group was randomly selected from a larger group of 430 individuals (210 women; age *M* = 23.45, SD = 5.61) based on their results on the CA questionnaire and a psychiatric disorder screening. Only subjects without current and lifetime diagnoses of selected psychiatric disorders [i.e., anxiety disorders including posttraumatic stress disorder (PTSD) and affective disorders] were included. Exclusion criteria were the presence of neurological disorders, traumatic brain injury, and addiction to alcohol, drugs or other psychoactive substances as well as any magnetic resonance imaging (MRI) contraindications. All participants were recruited *via* flyer advertisements, email advertisements. and in-class announcements at the University of Warsaw. A comparison of demographic characteristics and CA score between selected and non-selected individuals is shown in Table 1.

**Table 1:** Comparison of demographic characteristics and intensity of childhood adversity (CA) between selected and non-selected individuals.

	Selected individuals	Non-selected individuals	Statistics
Sex	F = 23; M = 25	F = 187; M = 195	χ^2^_(1, *N* = 430)_ = 0; *p* = 0.98
Age	M = 23.08; SD = 5.24	M = 23.5; SD = 5.66	*U* = 8387.5; *p* = 0.61
CQ score	M = 3.64; SD = 2.65	M = 2.76; SD = 2.57	*U* = 7239.5; *p* = 0.02

*CQ score—total CA score*.

There were no differences between groups in terms of sex and age. However, we identified significant differences in CA score—selected individuals reported more adverse experiences than non-selected individuals.

Subjects were paid 200 PLN (circa 50USD) for their participation in the study. Before consenting, each participant was given detailed information regarding the research goal and process. The Ethics Committee of the Faculty of Psychology of the University of Warsaw accepted the studied project. The study was carried out according to the Helsinki Declaration.

### Measures

Childhood adversities were measured with the use of a Polish version of the Childhood Questionnaire (CQ; Hardt et al., 2003). The CQ is a self-reported retrospective measure of the occurrence of 14 types of adverse experiences before the age of 14 (e.g., sexual abuse, physical abuse, poverty, the death of a parent). Some experiences were assessed separately for each parent, so the scale has a total of 18 possible experiences. Each item is answered with yes/no. In this study, we used the total CA score (CQ score), which is the sum of all adverse experiences indicated by the individual. The CQ score shows good internal consistency (Cronbach’s alpha = 0.71 in the current sample) and test-retest coefficients in the 0.80 range over a 2-year period.

The occurrence of psychiatric disorders was determined using the Composite International Diagnostic Interview-World Health Organization (CIDI-WHO; Kiejna et al., 2015). The CIDI-WHO is a structured interview which allows the assessment of symptoms of mental disorders. For the purposes of this study, only sections measuring affective and anxiety disorders were used. The criteria set by the ICD-10 were followed (World Health Organization, 1992).

The Polish version of the BIS/BAS Scales was used to measure temperament traits (Müller and Wytykowska, 2005). The BIS/BAS Scales comprises of a single 7-item scale designed to assess BIS features and three scales, Reward Responsivity (BAS-RR; 5 items), Drive (BAS-D; 4 items), and Fun Seeking (BAS-FS; 4-items), which assess different aspects of BAS functioning. Items are completed using a 4-point scale (from 1—disagree strongly to 4—agree strongly). The subscales demonstrate good internal reliability (0.78 for BIS, 0.65 for BAS-RR, 0.73 for BAS-D, and 0.65 for BAS-FS in the current sample).

### Neuroimaging Data Acquisition

Whole-brain imaging was performed with a 3-Tesla MRI scanner (Siemens Magnetom Trio TIM, Erlangen, Germany) equipped with 32-channel phased array head coil. Head movements were minimized with cushions placed around the participants’ heads. A set of high-resolution T1-weighted images (T1w) was acquired using the following acquisition parameters: TR: 2,530 ms, TE: 3.32 ms; flip angle: 7°. One dataset consisted of 176 sagittal images with an in-plane resolution of 1 mm^3^, field of view: 256 mm; slice thickness: 1 mm.

### Neuroimaging Data Analysis

Statistical Parametric Mapping (SPM12; Ashburner et al., 2014) running on MATLAB was used for data processing and statistical analyses. First, T1w structural images from single subjects were visually inspected according to Ashburner’s (2010) manual. The whole sample of collected images was suitable for further analyses. Images were automatically classified into gray matter, white matter, and cerebrospinal fluid using the “New Segmentation” tool based on a mixture of Gaussian models and tissue probability maps (Ashburner and Friston, 2005). The quality of segmented images was visually inspected. High-dimensional Diffeomorphic Anatomical Registration Through Exponentiated Lie Algebra (DARTEL; Ashburner, 2007) was used to create a study-specific template. Finally, images were normalized, modulated, and smoothed with an isotropic Gaussian kernel of 8 mm of full-width half maximum. Smoothed modulated normalized images were entered into a design matrix with age and total intracranial volumes as separate regressors. Analyses were computed in a single model with images from both women and men. Statistical inference was made by the whole-brain voxel-wise fitting of a general linear model, and statistical significance was corrected for multiple comparisons. An extent threshold of 20 voxels was set for all analyses based on prior neuropsychological VBM articles (e.g., Genschaft et al., 2013; Yin et al., 2013). For *a priori* hypotheses in pre-specified regions (right dlPFC, right hippocampus, right postcentral gyrus, left IPL, right and left cerebellum, and right and left fusiform gyrus), the threshold was set at *p* < 0.05 after Family-wise error correction for multiple comparisons in small volumes (small volume correction, SVC; Friston, 1997). The Montreal Neurological Institute coordinates were translated into Talairach space using GingerALE software (Eickhoff et al., 2012). Then, TalairachClient 2.4.2 was used to identify statistically significant clusters (Lancaster et al., 2000).

### Procedure

The CIDI-WHO, the BIS-BAS Scales, and the CQ were presented (in that order) to participants during the first session and MRI scanning was performed during the second session (approximately 6 months later).

### Statistical Analysis

Spearman’s rho was used to evaluate simple relationships of the number of childhood adversities with BIS and BAS activities. The data were analyzed using SPSS software.

## Results

**Figure 1:**
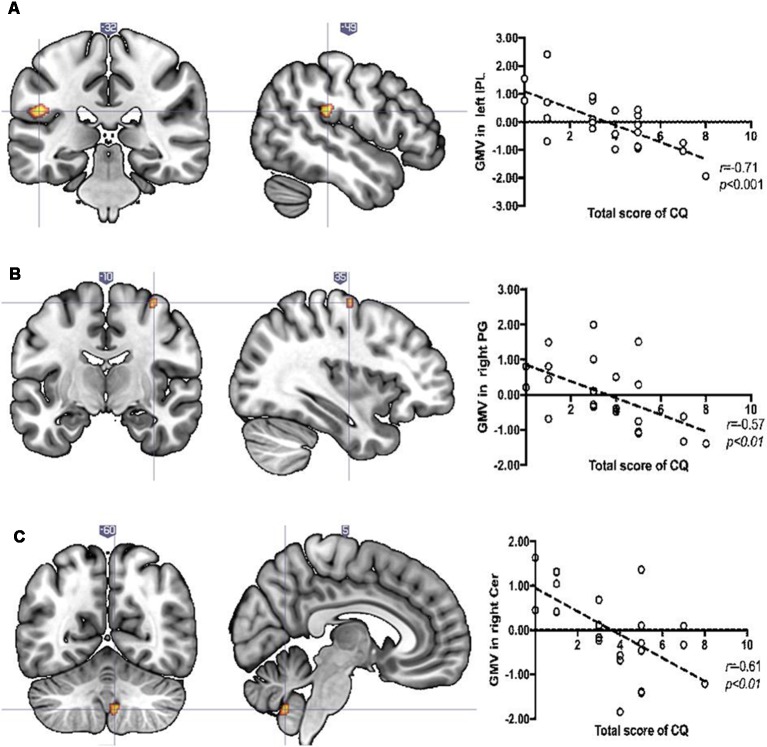
Significant decrease in regional gray matter volume (GMV) related to Childhood Questionnaire (CQ) score in the **(A)** left inferior parietal lobule (IPL), **(B)** right precentral gyrus, and **(C)** right cerebellum in the female sample. Scatterplots illustrate relationships between CQ score and GMV (for display purposes only). The *x*-axis of the scatterplot represents standardized residuals of GMV and the *y-axis* represents the total score of CQ. For standardized residual measures, age and total intracranial volumes were regressed out.

**Figure 2:**
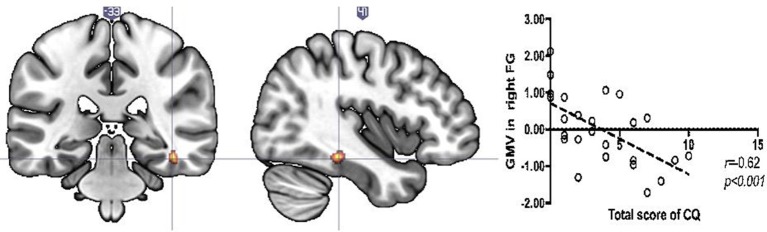
Significant decrease in regional GMV related to childhood adversity (CA) score in right fusiform gyrus in the male sample. Scatterplot illustrates the relationship between CQ score and GMV (for display purposes only). The *x*-axis of the scatterplot represents standardized residuals of GMV and the *y-axis* represents the total score of CQ. For standardized residual measures, age and total intracranial volumes were regressed out.

There were no significant differences between women and men in terms of age (*U* = 208.5, *p* = 0.1) or total CA score (*U* = 280, *p* = 0.88). Relationships between CQ score and GMV were tested across the entire sample as well as the interaction effect. A significant interaction was found in left inferior parietal lobule/parietal operculum (left IPL/OP; *F* = 20.67; 168 voxels; x y z: −51 −33 21) and right fusiform gyrus (right FG; *T* = 4.09, 43 voxels, x y z: 42 −33 −16). The former was driven by a negative association between CQ score and GMVs in women (left IPL; *T* = 4.14, 130 voxels, x y z: −51, −33, 23), but not in men, while the latter was driven by the negative association between CQ score and GMV only in men (right FG; *T* = 4.09, 43 voxels, x y z: 42 −33 −16). Additionally, for women, we found a negative association between CQ score and GMVs in right cerebellum (right Cer; *T* = 3.65, 43 voxels, x y z: 6 −60 −45) and right precentral gyrus (right PG; *T* = 3.5, 20 voxels, x y z: 34 −9 63). The results of neuroimaging analyses are shown in Figures 1, 2.

Separate correlation analyses were performed for female and male subsamples to test relationships between CQ score and BIS and BAS activities. The results are shown in Table 2.

**Table 2:** Correlation coefficients (Spearman’s rho) between the number of childhood adversities and temperament traits among females and males.

	BIS	BAS	BAS-RR	BAS-D	BAS-FS
Female sample	0.56**	−0.37	−0.65**	−0.12	−0.07
Male sample	−0.33	−0.37	−0.37	−0.14	0.24

**p < 0.01; BIS—BIS activity; BAS—BAS activity; BAS-RR—BAS Reward Responsiveness; BAS-D—BAS Drive; BAS-FS—BAS Fun Seeking.

In the female subsample, we found a positive relationship between CQ score and BIS activity and, unexpectedly, a negative relation between CQ score and BAS Reward Responsiveness. In the male subsample, there was no significant relation between CQ score and temperament.

## Discussion

The problem of the relationship between CA and GMV has been the subject of many studies. However, in previous analyses, the impact of potential confounding variables such as sex and the occurrence of the mental health problems was underestimated. The primary aim of the current study was to understand sex-specific relations between CA and GMV in a group of participants without anxiety or affective disorders. First of all, we did not confirm the results of the meta-analysis of Paquola et al. (2016). They identified two regions related to CA (right dlPFC and right postcentral gyrus) for cohorts with more female participants. In our opinion, it is hard to compare the results of our study to the results of the work of Paquola et al. (2016) due to differences in the analytic process (for example, in their meta-analysis sex and the occurrence of mental problems were regressed on GMVs in separate steps). However, we cannot exclude that our failure to replicate the results of Paquola et al. (2016) is related to some limitations of our study, which are described in the final section of the discussion. Interestingly, we identified sex-specific associations which were detected in previous studies on samples of both sexes between the CA score (CQ score) and GMV in several regions of the brain. In the female group, we found a negative relationship between CQ score and GMV in the left IPL. Several studies highlight the role of this part of the parietal cortex in negative emotional processing and modulation of arousal. The meta-analysis of Fusar-Poli et al. (2009) revealed increased activation in this structure when processing fearful and disgusted faces as compared to baseline among healthy subjects. What is more, a meta-analysis by Etkin and Wager (2007) found hyperactivation of the left IPL in patients with PTSD when processing emotional stimuli as compared to neutral ones. A recent analysis (Cheng et al., 2015) identified reduced GMV in the left IPL among patients with PTSD when compared to healthy controls. Interestingly, Bramen et al. (2012) revealed a negative relationship between cortical thickness in this region and the level of circulating testosterone in girls but not in boys. Data indicate that the functioning of the hypothalamic-pituitary-adrenal (HPA) and the hypothalamic-pituitary-gonadal (HPG) axes is linked during adolescence and that cortisol–testosterone coupling is influenced by early life stress. This effect seems to be unique to girls (Ruttle et al., 2015).

The right precentral gyrus is another structure which was related to CQ score in females in our sample. This region is implicated in the regulation of emotion (Seo et al., 2013) and self-evaluation, including body image (Morita et al., 2008). Our finding is supported by a study demonstrating that the right precentral gyrus volume was negatively related to CA in a group of adolescents with alcohol-use disorder (Brooks et al., 2014). Interestingly, the results of a recent study by Hardee et al. (2017), performed on a group of children and adolescents with high levels of parental substance use disorders, strengthen the sex-specific direction of our finding. The authors of this longitudinal functional MRI (fMRI) study revealed age-related sex differences in the activity of the right precentral gyrus when processing negative vs. neutral words: within the female subsample, the response was stable over time, whereas it became diminished among males.

Low cerebellar volumes are associated with maltreatment in early childhood (De Bellis and Kuchibhatla, 2006) as well as other types of trauma (Baldaçara et al., 2011). It is well established that the cerebellum plays an important role in emotional and cognitive development (Schmahmann, 2010). Interestingly, in a recent study on maltreated youth with PTSD (Crozier et al., 2014), sex differences were found in the activation of the cerebellum during the execution of an emotional oddball task.

The left cerebellum and right precentral gyrus are implicated in reward processing, particularly food intake and anticipated intake or disturbances thereof in eating disorders (e.g., Sanders et al., 2015). The animal model of neonatal isolation revealed an enhancing effect of early life stress on response to foods in females but not males (Kosten et al., 2006). The authors indicated that this finding might, in part, reflect variability in the ventral striatal dopamine system. A growing body of evidence suggests that striatal dopamine plays a crucial role in modulating the function of, among others, the IPL and precentral gyrus (Albrecht et al., 2014). Moreover, cerebellum and basal ganglia interactions were shown to contribute to reward-related learning (Swain et al., 2011), in which dopamine plays a crucial role (Hyman et al., 2006). As we emphasized above, cortisol–testosterone coupling may impact brain maturation processes in a specific manner. Undoubtedly, dopamine neurotransmission is one of the brain mechanisms which is affected by interactions between stress and sex hormones (Sinclair et al., 2014). Thus, one may speculate that our findings reflect a sex-specific effect of early stress on brain structures involved in behavioral problems linked to impaired reward processing. It should be underlined that our sample consisted only of participants without a diagnosis of anxiety or affective disorders. Hence, structural differences related to CA revealed in our study may be treated as a potential endophenotype of disorders which are correlated with behavioral disinhibition.

On the other hand, in the male group, we found a negative relationship between CQ score and GMV in the right fusiform gyrus. This region was found to be specifically activated during face processing (Grill-Spector et al., 2004), whereas sex affects the amygdala-fusiform gyrus connection during stress, with males exhibiting reduced coordination of these two structures while viewing emotionally arousing stimuli (Mather et al., 2010). Our findings seem to be consistent with the results of the study by Brooks et al. (2015). They found a negative relationship between having experienced early-life adversity, measured by the Childhood Trauma Questionnaire, and GMV in the right fusiform gyrus. What is more, in the study of Cheng et al. (2015), PTSD patients showed a reduction in this area compared to healthy controls. However, one must remember that research of Brooks et al. (2015) and Cheng et al. (2015) utilized psychiatric cohorts, while we used a community sample with subjects without a mental health diagnosis. We also keep in mind a notion that a region referred in one study may not be the same in another study. As we stated above, we believe that structural differences related to CA that we revealed in our study may be treated as potential endophenotypes of the psychiatric condition. Hence, the results of previous studies on CA-GMV relationship using psychiatric cohorts may be used as an indirect confirmation of our findings.

We were only able to partially confirm the hypothesis regarding the relationship between CA and temperament. As we hypothesized, we found sex-specific relationships between CQ score and BIS activity among females. We also unexpectedly found a negative relationship between CA and BAS Reward Responsiveness in the female subsample. Moreover, we did not find any relationship between CQ score and temperament in the male sample. The sex-specific correlation between CA and temperament is supported by the results of the study of Sudbrack et al. (2015). Our findings also are consistent with data on the sex-differentiated impact of early life stress on the development of psychopathology. One might speculate that the negative relationship between CQ score and Reward Responsiveness may reflect the complex influence of BIS and BAS on behavior. Contemporary conceptualizations of Gray’s concepts include the joint action of both systems in shaping one’s personality (Corr, 2004). Overall, our findings seem to be supported by the results of a study on the neuroanatomical basis of the BIS/BAS systems (Li Y. et al., 2014). The authors showed sex-specific patterns of relationships between temperament and GMV in brain areas associated with the processing of negative emotions and reward-related information. However, one has to remember that the method of analysis, which we used to study relationships between CQ score and temperament, is not a direct way of comparing both sexes. Due to this caution is needed while interpreting the results of our study in terms of sex differences.

Although our study brings novel insights to the sex-specific relationship between CA and differences in GMV, it is not free from potential limitations. First, our analysis is based on a retrospective self-report measure of CA. Although the CQ has been extensively validated (Hardt and Rutter, 2004), its reliance on retrospective recall of adversity opens the possibility that recall bias may have confounded our results. Second, our sample consisted mostly of university students, so the results of this study may not be generalizable to all population groups. Third, although we used a structured interview to select participants without signs of psychiatric disorders, we only used parts measuring anxiety and affective disorders. Therefore we cannot exclude that the results of our study are biased by the impact of non-diagnosed mental health problems. Fourth, we performed MRI scanning approximately 6 months after psychiatric disorders’ screening. Thus, we cannot exclude that mental health status of participants changed. Fifth, we did not include the measure of the timing of specific CA in our analysis. Teicher et al. (2006) emphasized that the timing of CA may impact some brain regions undergoing distinct growth spurts at the time. Thus, the lack of such a measure in our study is its severe limitation. Finally, given its relatively small sample size and related to this fact the use of non-parametric statistical methods, the results of our study should be treated cautiously.

Notwithstanding these limitations, this study has several aspects that strengthen its conclusions. Firstly, as we mentioned above, our sample consisted only of mentally healthy participants, thus removing potential confounding variables associated with pathological processes in the brain. Secondly, this study fills a gap in the lack of data on sex-specific relations between CA and GMV. Finally, taking into account the two advantages mentioned above, our study replicates some previously published findings on the impact of early life stress on brain structure.

In summary, we found a sex-specific relationship between the CA score and GMV. Despite the fact that the study included subjects without a mental health diagnosis, its results indicate that early stressors may leave permanent scars on brain structure. Additional work is needed for a more comprehensive understanding of the mechanisms underlying sex differences in this relationship.

## Ethics Statement

The protocol of the study was approved by the Ethics Committee of the Faculty of Psychology at the University of Warsaw. All subjects gave written informed consent in accordance with the Declaration of Helsinki.

## Author Contributions

WD: plan and organization of the research, data analysis, and manuscript writing. KJ and AM: data analysis and manuscript writing.

## Conflict of Interest Statement

The authors declare that the research was conducted in the absence of any commercial or financial relationships that could be construed as a potential conflict of interest.
